# Relationship between metabolically healthy obesity and the development of hypertension: a nationwide population-based study

**DOI:** 10.1186/s13098-022-00917-7

**Published:** 2022-10-13

**Authors:** Yue Yuan, Wei Sun, Xiangqing Kong

**Affiliations:** 1 Department of Cardiology, The First Affiliated Hospital of Nanjing Medical University, 300 Guangzhou Road, Nanjing, 210029 People’s Republic of China; 2 Cardiology, Nanjing Medical University, Nanjing, People’s Republic of China

**Keywords:** Metabolically healthy obesity, Hypertension, Abdominal obesity, Epidemiology, General obesity

## Abstract

**Background:**

Metabolically healthy obesity (MHO), has been recognized as a transient phenotype with few cardiometabolic diseases; however, little is known regarding the development of hypertension in subjects with an absence of cardiometabolic abnormalities and general obesity evaluated by body mass index (BMI) or abdominal obesity evaluated by waist circumference (WC).

**Methods:**

A total of 4764 participants were enrolled from the China Health and Nutrition Survey and followed up from 2009 to 2015, whose fasting blood samples were collected in 2009. Obesity was classified as abdominal obesity (WC ≥ 90 cm in men and ≥ 80 cm in women) and general obesity (BMI ≥ 25.0 kg/m^2^). Logistic regression was used to analyze the relationship between MHO and prehypertension (120 < SBP < 140 mmHg or 80 < DBP < 90 mmHg) and hypertension (SBP ≥ 140 or DBP ≥ 90 mmHg). The age- and sex-specific impacts were further analyzed.

**Results:**

There were 412 (37.9%) participants with prehypertension and 446 (41.0%) participants with hypertension and metabolically healthy abdominal obesity (MHAO). The participants with the MHAO phenotype had significantly higher risks of prehypertension [odds ratio (OR) = 1.89 (1.51–2.36), *p* < 0.001] and hypertension [OR = 2.58 (2.02–3.30), *p* < 0.001] than those metabolically healthy but without abdominal obesity. Similar associations were observed in the subjects with metabolically healthy general obesity (MHGO) phenotype, particularly those aged under 64 years. Men with the MHAO phenotype seemed to have higher risks of prehypertension [2.42 (1.52–3.86) in men vs. 1.76 (1.36–2.29) in women] and hypertension [3.80 (2.38–6.06) in men vs. 2.22 (1.64-3.00) in women] than women, when compared with those metabolically healthy but without abdominal obesity.

**Conclusion:**

The MHO phenotype, regardless of the presence of general or abdominal obesity, showed a worse effect on the development of prehypertension and hypertension, particularly in young adults. Abdominal adiposity with a healthy metabolic state is significantly associated with incident hypertension in both men and women. These findings can guide the establishment of risk-stratified obesity treatments.

**Supplementary Information:**

The online version contains supplementary material available at 10.1186/s13098-022-00917-7.

## Introduction

The prevalence of overweight and obesity has increased over the past forty years as a result of increased intake of high-fat diets and physical inactivity [1, 2], amplifying the burden of subsequent cardiovascular diseases (CVDs) [3]. The accurate classification and management of adiposity and metabolic states has become a mainstream trend. It is generally accepted that obesity is a remarkably heterogeneous status with varying cardiovascular and metabolic manifestations, which are characterized by alteration in fat metabolism through lipid and should be classified into general and abdominal obesity accumulation [4, 5]. Metabolically healthy general obesity (MHGO) refers to obesity (defined by body mass index [BMI]) without cardiometabolic abnormality (CA) [6, 7]. Age- and sex-dependent prevalence of MHGO varies from 10 to 40% across cohorts [8]. Our previous study showed that MHGO phenotype did not significantly increase arterial stiffness, compared with the metabolically healthy lean phenotype [9]. In contrast, another study showed that individuals with MHGO phenotype had higher risks of coronary heart disease and heart failure than those with metabolically healthy normal weight [10]. The relationship between MHO and subsequent CVDs is still controversial .

Hypertension is a prominent risk factor for cardiovascular death [11]. The relationship between MHO and hypertension has not been fully demonstrated, due to its distinct classifications of obesity and metabolic abnormalities. A recent study has reported that MHO phonotype, regardless of the presence of general or abnormal adiposity, is positively associated with the risk of hypertension among individuals living in rural areas in centra China [12]. A meta-analysis of eight Asian prospective cohort studies has reported a significant positive association between MHGO and the risk of hypertension (pooled effect size: 1.54, 95% CI 1.48–1.55) [13]. Previous studies have indicated that the association of obesity phenotype with incident hypertension accompanied by pathological inflammation is sex- or age-specific [14, 15]. Oxidative stress resulting from abnormal lipid and glucose metabolism increases the levels of pro-inflammatory proteins and inflammatory cytokines, thus driving the development of hypertension [16].

In this study, we aimed to explore the value of the combination of metabolic state (general and abdominal adiposity) and obesity in predicting the development of prehypertension and hypertension. We also aimed to identify the sex- and age-specific relationships between elevated blood pressure (BP) and MHGO and metabolically healthy abdominal obesity (MHAO). It can be meaningful to better understand of whether and how different obesity treatment strategies may promote individual treatment decisions based on the MHGO and MHAO phenotypes.

## Methods

### Study cohort

**Fig. 1:**
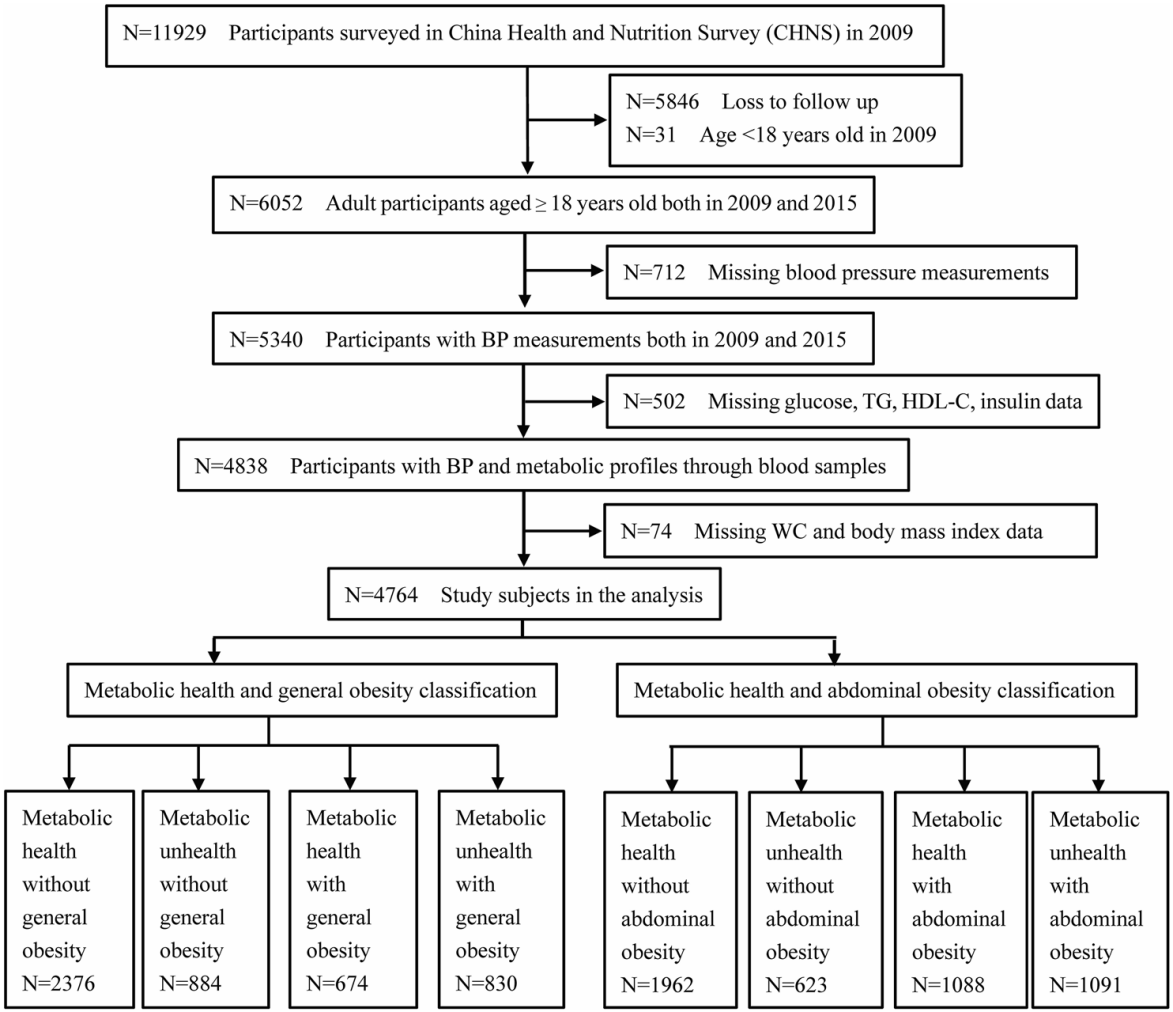
Flow chart of study participants enrolled from the China Health and Nutrition Survey cohort (n = 4764). (BP: blood pressure; TG: triglycerides; HDL-C: high-density lipoprotein cholesterol; WC: waist circumference)

The China Health and Nutrition Survey (CHNS) was initiated in 1989 and involved 11,929 participants living in cities and rural areas in nine provinces, including Liaoning, Heilongjiang, Jiangsu, Shandong, Henan, Hubei, Hunan, Guangxi, and Guizhou. This ongoing observational cohort study is representative of Chinese participants due to its large scale and standard conduction. The CHNS is an ongoing, open, prospective cohort study in China, and ten CHNS rounds have been completed, respectively in 1989, 1991, 1993, 1997, 2000, 2004, 2006, 2009, 2011 and 2015. Participant enrollment and information collection have been previously described [17]. The survey materials and acknowledgements can be found on the website (http://www.cpc.unc.edu/projects/china). The first blood sample collection was conducted on a large scale in 2009. We excluded individuals who aged < 18 years (N = 31); lost to follow up in 2015 (N = 5846); lacked BP data in 2009 and 2015 (N = 712); missed fasting plasma glucose (FPG), triglyceride (TG), high-density lipoprotein cholesterol (HDL-C), insulin in blood samples (N = 502); and lacked waist circumference (WC) and body mass index (BMI) (N = 74). The flow chart is shown in Fig. 1. Finally, 4764 participants (2145 males and 2619 females) were included in the analysis. The CHNS study was approved by the institutional review committees of the National Institute of Nutrition and Food Safety, the University of North Carolina at Chapel Hill (No. 201524-1), and the China-Japan Friendship Hospital, the Ministry of Health, the Chinese Center for Disease Control and Prevention (2,015,017). The protocols were in accordance with relevant guidelines and regulations.

### General examinations

Clinical demographic information, including sex, age, community type, marital status, education level, smoking habits, alcohol consumption and histories of hypertension, diabetes mellitus and related medical treatment, was obtained by trained staff through standardized self-questionnaires. Physical examinations, including height, weight, WC and hip circumference, were performed repeatedly by trained investigators using calibrated beam scales. WC was measured at a midway point between the lowest rib and the iliac crest by a nonelastic tape. BMI was defined as kg/m^2^ at study entry and calculated as body weight (kg) divided by the square of height (m^2^) to evaluate the general adiposity state. The subjects were asked to rest for at least 10 min before BP measurement. Using standard mercury sphygmomanometers, the trained staff measured the systolic BP (SBP) and diastolic BP (DBP) three times on the right arms of the seated subject hearing Phase I and V Korotkoff sounds. BP was measured three times on the right upper arm in a seated position after a 5-minute rest, with a 2-minute interval between measurements. The average of three BP values was used in the analyses. The mean arterial pressure (MAP) was calculated by the formula MAP = 1/3 SBP + 2/3 DBP (in mmHg).

Fasting blood samples were collected by trained nurses according to the standard protocol and guidelines, and transferred to a national central laboratory in Beijing (Medical Laboratory Accreditation Citation certificate: ISO 15189:2007) [18]. The biochemical marker information and measurement methods are provided on the website (https://www.cpc.unc.edu/projects/china/data/datasets/biomarker-data). Biochemical markers, including HDL-C, low-density lipoprotein cholesterol (LDL-C), total cholesterol (TC), TG, urea, serum uric acid, serum creatinine, total protein, albumin and alanine aminotransferase levels, were measured by an automatic clinical chemistry analyzer (Hitachi 7600 D, Japan). FPG was measured by the GOD-PAP method (Randox Laboratories Ltd, UK). Apolipoprotein A (ApoA) and apolipoprotein B (ApoB) were measured by immunoturbidimetric methods (Randox Laboratories Ltd, UK), and insulin levels were measured by a radioimmunology assay (Gamma counter XH-6020, Beijing, China).

### Definitions of hypertension and metabolic health and obesity

Subjects with an SBP ≥ 140 mmHg or a DBP ≥ 90 mmHg and those who were receiving treatment for hypertension were defined as having hypertension in this study [19]. Subjects with 120 < SBP < 140mmHg or 80 < DBP < 90mmHg were defined as having prehypertension [20]. BMI was used to assess the general adiposity in subjects with a BMI of ≥ 25.0 kg/m^2^ based on the diagnostic criteria for Asian people released by World Health Organization Western Pacific Region [21]. WC was used to assess abdominal adiposity, with ≥ 90 cm for men and ≥ 80 cm for women [22]. The following National Cholesterol Education Program Adult Treatment Panel III (NCEP ATP III) criteria were used to define CAs in the current study [23]: (1) an elevated SBP/DBP of ≥ 130/85 mmHg or on antihypertensive treatment; (2) a high FPG level of ≥ 100 mg/dL (5.6 mmol/L) or on hypoglycemic treatment; (3) a high TG level of ≥ 1.7 mmol/L or on lipid-lowering therapy; and (4) a low HDL-C level (< 1.04 mmol/L in men and < 1.29 mmol/L in women) or on lipid-lowering medications. The combination of CAs with general adiposity was classified into: (1) metabolically healthy without general obesity: a BMI < 25 kg/m^2^ and < 2 CAs; (2) metabolically unhealthy without general obesity: a BMI < 25 kg/m^2^ and ≥ 2 CAs; (3) MHGO: a BMI ≥ 25 kg/m^2^ and < 2 CAs; and (4) metabolically unhealthy general obesity (MUGO): a BMI ≥ 25 kg/m^2^ and ≥ 2 CAs [9].

The combination of CAs with abdominal adiposity was classified into: (1) metabolically healthy without abdominal obesity, a WC < 90 cm in men and < 80 cm in women and < 2 CAs; (2) metabolically unhealthy without abdominal obesity, a WC < 90 cm in men and < 80 cm in women and ≥ 2 CAs; (3) MHAO, a WC ≥ 90 cm in men and ≥ 80 cm in women and < 2 CAs; and (4) metabolically unhealthy abdominal obesity (MUAO), a WC ≥ 90 cm in men and ≥ 80 cm in women and ≥ 2 CAs [22].

### Statistical analysis

In the current study, we used SPSS version 16.0 for Mac (SPSS Inc., Chicago, IL, USA) to conduct all analyses. The demographic characteristics were compared between the subjects with different metabolic states and obesity phenotypes. The continuous variables were shown as means ± standard deviations (SDs), and compared via one-way ANOVA with Holm-Sidak pos hoc test if the data were normally distributed. The Mann–Whitney U test was performed if the data were not normally distributed. Categorical data were expressed as percentages, and the comparison of qualitative variables was performed via χ² tests. Logistic regression was used to derive the odds ratios (ORs) and 95% confidence intervals (CIs) to determine the 6-year risk of distinct metabolic obesity states for the development of hypertension. The full adjustment model involved age, sex, smoking habits, alcohol consumption, community type, marital status and education level, and urea, serum uric acid, serum creatinine, FPG, TC, TG, HDL-C, LDL-C, white blood cell (WBC), red blood cell (RBC), platelet, hemoglobin A1c, hemoglobin, total protein, albumin, alanine aminotransferase, Apo-A, and Apo-B levels. We further analyzed the sex-specific and age-specific relationships between incident hypertension and metabolic obesity state. Statistical significance was determined with a two-sided *p* < 0.05.

## Results

Table 1 shows the comparison of clinical demographic characteristics and cardiometabolic risks among the four metabolic states combined with abdominal obesity phenotype. The levels of BMI, WC, hip circumference, SBP, DBP, MAP, serum urea, uric acid, creatinine, HDL-C, LDL-C, TC, TG, insulin, FPG, WBCs, RBCs, platelets, hemoglobin A1c, hemoglobin, total protein, albumin, alanine aminotransferase, Apo-A, and Apo-B at baseline differed significantly according to metabolically healthy and abdominal obesity status (Table 1, all *p* values < 0.05). The percentages of individuals who smoked, consumed alcohol, had hypertension and had diabetes varied significantly across the four groups (all *p* values < 0.05). Males seemed to be more likely to be prehypertensive and hypertensive (*p* value < 0.05), as shown in Additional file 1: Table S1. In 2015, the subjects with prehypertension and hypertension had significantly higher BMIs, WCs and hip circumferences than subjects with normal blood pressure (Additional file 1: Table S1, *p* value < 0.05).

**Table 1:** Clinical characteristics of the study participants according to WC and metabolic status in the cohort study

Characteristics	N	Metabolically healthy without abdominal obesity	Metabolically unhealthy without abdominal obesity	Metabolically healthy with abdominal obesity	Metabolically unhealthy with abdominal obesity	*P*
N	4764	1962	623	1088	1091	−
Gender (%)	4764					< 0.001
Male		1085 (55.3)	409 (65.7)	269 (24.7)	382 (35.0)	
Parameter in 2009						
Age, yr	4764	49.00 (39.00–59.00)	53.00 (44.00–61.00)	51.00 (43.00–60.00)	55.00 (46.00–62.00)	< 0.001
Living area (%)	4764					0.121
Urban		560 (28.5)	204 (32.7)	301 (27.7)	328 (30.1)	
Rural		1402 (71.5)	419 (67.3)	787 (72.3)	763 (69.9)	
Marital status (%)	4764					< 0.001
Married		1734 (88.4)	569 (97.6)	992 (91.2)	955 (87.5)	
Divorced		20 (1.0)	5 (0.9)	10 (0.9)	13 (1.2)	
Unmarried or other		208 (10.6)	49 (1.5)	86 (7.9)	123 (11.3)	
Education year, yr	4761	7.30 ± 4.40	7.29 ± 4.52	6.48 ± 4.62	6.27 ± 4.65	< 0.001
Smoking (%)	4764	748 (38.1)	280 (44.9)	173 (15.9)	265 (24.3)	< 0.001
Drinking (%)	4764	763 (38.9)	266 (42.7)	245 (22.5)	300 (27.5)	< 0.001
Hypertension (%)	4764	117 (6.0)	90 (14.4)	123 (11.3)	288 (26.4)	< 0.001
Diabetes (%)	4764	17 (0.9)	22 (3.5)	16 (1.5)	72 (6.6)	< 0.001
BMI, kg/m^2^	4764	21.38 ± 2.40	22.63 ± 2.48	25.02 ± 2.86	26.50 ± 3.22	< 0.001
WC, cm	4764	76.00 (71.00–80.00)	79.00 (74.00-84.40)	89.50(83.28-94.00)	92.00(87.00–98.00)	< 0.001
Hip, cm	4728	90.00 (86.35–94.00)	92.00 (88.00–96.00)	98.00 (94.00-102.80)	100.00(96.00-105.00)	< 0.001
SBP, mm Hg	4764	118.41 ± 15.84	130.33 ± 18.62	123.97 ± 17.21	135.87 ± 20.06	< 0.001
DBP, mm Hg	4764	76.82 ± 10.12	84.65 ± 10.75	80.58 ± 10.62	87.14 ± 11.20	< 0.001
MAP, mm Hg	4764	90.68 ± 11.11	99.87 ± 12.07	95.04 ± 12.00	103.38 ± 12.75	< 0.001
Urea, mmol/L	4764	5.47 ± 1.66	5.66 ± 1.48	5.46 ± 1.44	5.57 ± 1.48	0.028
Serum uric acid, mmol/L	4764	277.00 (228.00-334.00)	337.00 (270.00–412.00)	263.00 (219.25–315.00)	329.00 (271.00-394.00)	< 0.001
Serum creatinine, mmol/L	4764	86.00 (77.00–96.00)	90.00 (80.00–100.00)	80.00 (73.00–89.00)	83.00 (75.00–94.00)	< 0.001
HDL-C, mmol/L	4764	1.58 ± 0.45	1.27 ± 0.41	1.52 ± 0.38	1.21 ± 0.37	< 0.001
LDL-C, mmol/L	4763	2.86 ± 0.85	2.90 ± 1.04	3.15 ± 0.85	3.12 ± 1.10	< 0.001
TC, mmol/L	4764	4.60 (4.03–5.19)	4.89 (4.30–5.60)	4.85 (4.23–5.49)	5.08 (4.49–5.81)	< 0.001
Triglycerides, mmol/L	4764	0.97 (0.71–1.29)	2.08 (1.58–3.10)	1.12 (0.82–1.44)	2.29 (1.67–3.14)	< 0.001
Insulin, IU/mL	4764	8.57 (6.28–11.83)	11.59 (8.19–17.07)	10.25 (7.49–14.52)	13.82 (9.84–20.59)	< 0.001
White blood cell count, 10^9^/L	4751	6.06 ± 1.94	6.46 ± 1.72	6.04 ± 1.66	6.58 ± 2.13	< 0.001
Red blood cell count, 10^12^/L	4726	4.69 ± 0.70	4.82 ± 0.70	4.61 ± 0.64	4.74 ± 0.65	< 0.001
Platelet count, 10^9^/L	4748	208.00 (164.00–250.00)	209.5 0 (166.75–258.00)	215.00 (172.00–258.00)	210.00 (169.00–253.00)	0.253
Hemoglobin A1c, %	4741	5.41 ± 0.62	5.74 ± 1.46	5.59 ± 0.64	6.00 ± 1.11	< 0.001
Hemoglobin, g/L	4750	140.00 (127.00-152.00)	147.00 (133.00–159.00)	137.00 (127.00–148.00)	141.00 (130.00–155.00)	< 0.001
Total protein, g/L	4764	76.60 (73.40–80.00)	77.00 (73.50–80.70)	77.30 (74.03–80.20)	77.40 (74.10–81.00)	< 0.001
Albumin, g/L	4764	46.90 (44.90–49.10)	47.50 (45.60–50.10)	47.10 (45.20–49.00)	47.70 (45.70–49.80)	< 0.001
Fasting plasma glucose, mmol/L	4764	4.90 (4.56–5.27)	5.67 (5.01–6.24)	5.01 (4.70–5.36)	5.68 (5.18–6.43)	< 0.001
Alanine Aminotransferase, U/L	4763	16.00 (12.00–23.00)	20.00 (15.00–30.00)	18.00 (14.00–26.00)	23.00 (16.00–32.00)	< 0.001
Apolipoprotein A, g/L	4764	114.00 (100.00–134.00)	106.00 (90.00–129.00)	112.00 (97.25–130.00)	102.00 (88.00-120.00)	< 0.001
Apolipoprotein B, g/L	4764	81.00 (67.00–96.00)	94.00 (77.00–113.00)	89.00 (75.00–106.75)	102.00 (84.00-120.00)	< 0.001
HOMA-IR	4764	1.86 (1.31–2.58)	2.96 (1.99–4.66)	2.26 (1.63–3.32)	3.51 (2.37–5.94)	< 0.001
Parameter in 2015						
SBP in 2015, mm Hg	4764	126.04 ± 18.20	135.91 ± 19.54	133.05 ± 18.10	139.21 ± 20.22	< 0.001
DBP in 2015, mm Hg	4764	79.64 ± 10.29	83.72 ± 10.70	82.89 ± 10.53	85.69 ± 11.58	< 0.001
MAP in 2015, mm Hg	4764	95.11 ± 11.78	100.45 ± 12.34	99.27 ± 11.79	104.53 ± 12.94	< 0.001
BMI in 2015, kg/m2	4729	22.26 ± 2.82	23.40 ± 3.07	25.68 ± 3.28	26.67 ± 3.49	< 0.001
WC in 2015, cm	4736	80.00 (73.65–86.00)	83.80 (78.00–90.00)	88.00 (82.00–94.28)	93.00 (86.00–99.00)	< 0.001
Hip in 2015, cm	4735	92.00 (88.00–96.00)	94.00 (89.00–98.55)	98.00 (93.00–103.00)	100.00 (95.00–105.00)	< 0.001

WC: waist circumference; BMI, body mass index; SBP: systolic blood pressure; DBP: diastolic blood pressure; MAP: mean arterial pressure; HDL-C: high-density lipoprotein cholesterol; LDL-C: low-density lipoprotein cholesterol; TC: total cholesterol; HOMA-IR: homeostasis model assessment of insulin resistance; Non-normally distributed variables are expressed as the median (interquartile range). All other values are expressed as mean ± SD or n, %

### Predictive role of metabolic obesity phenotypes and elevated BP

Tables 2 and 3 present the incidences of prehypertension and hypertension predicted by metabolic abdominal and general obesity, respectively. To be specific, 412 (37.9%) participants with prehypertension and 446 (41.0%) participants with hypertension demonstrated MHAO. After full adjustment, the subjects with MHAO had significantly higher risks of prehypertension [OR = 1.89 (1.51–2.36), *p* < 0.001] and hypertension [OR = 2.58 (2.02–3.30), *p* < 0.001] than subjects with metabolically healthy but without abdominal obesity phenotype (Table 2). A similar relationship existed between metabolically healthy without general obesity and the risks of prehypertension and hypertension (Table 3).

**Table 2:** Adjusted odds ratios and 95% confidence intervals of the association of metabolic health and obesity with prehypertension and hypertension

BMI and metabolic status	No. with outcomes (%)	Mode 1		Model 2		Model 3	
		OR (95% CI)	*P* value	OR (95% CI)	*P* value	OR (95% CI)	*P* value
Prehypertension							
Metabolically healthy without general obesity	881 (37.1)	–	–	–	–	–	–
Metabolically unhealthy without general obesity	294 (33.3)	1.56 (1.25–1.94)	**< 0.001**	1.57 (1.26–1.96)	**< 0.001**	1.41 (1.06–1.88)	**0.017**
Metabolically healthy with general obesity	235 (34.9)	1.85 (1.45–2.36)	**< 0.001**	1.90 (1.48–2.42)	**< 0.001**	1.70 (1.31–2.21)	**< 0.001**
Metabolically unhealthy with general obesity	221 (26.6)	2.04 (1.57–2.67)	**< 0.001**	2.08 (1.60–2.72)	**< 0.001**	1.49 (1.04–2.14)	**0.032**
Hypertension							
Metabolically healthy without general obesity	691 (29.1)	–	–	–	–	–	–
Metabolically unhealthy without general obesity	432 (48.9)	2.68 (2.14–3.36)	**< 0.001**	2.79 (2.22–3.51)	**< 0.001**	2.46 (1.82–3.32)	**< 0.001**
Metabolically healthy with general obesity	318 (47.2)	3.40 (2.64–4.37)	**< 0.001**	3.40 (2.64–4.38)	**< 0.001**	2.92 (2.22–3.83)	**< 0.001**
Metabolically unhealthy with general obesity	518 (62.4)	6.01 (4.65–7.77)	**< 0.001**	6.04 (4.67–7.82)	**< 0.001**	4.48 (3.13–6.42)	**< 0.001**

BMI: body mass index; ORs: odds ratios

Metabolically healthy without general obesity was the reference group

Model 1: adjusted for age and sex;

Model 2: based on model 1 and smoke habits, alcohol consumption, community type, married status and education years

Model 3: based on model 2 and further adjusted for urea, serum uric acid, serum creatinine, fasting plasma glucose, total cholesterol, triglyceride, high-density lipoprotein cholesterol, low-density lipoprotein cholesterol, white blood cell count, red blood cell count, platelet count, hemoglobin A1c, hemoglobin, total protein, albumin, alanine aminotransferase, apolipoprotein A, apolipoprotein B

Significant values was in bold

**Table 3:** Adjusted odds ratios and 95% confidence intervals of the association of metabolic health and abdominal obesity with prehypertension and hypertension

WC and metabolic status	No. with outcomes (%)	Mode 1		Model 2		Model 3	
		OR (95% CI)	*P* value	OR (95% CI)	*P* value	OR (95% CI)	*P* value
Prehypertension							
Metabolically healthy without abdominal obesity	701 (35.9)	–	–	–	–	–	–
Metabolically unhealthy without abdominal obesity	209 (33.5)	1.65 (1.27–2.13)	**< 0.001**	1.68 (1.30–2.17)	**< 0.001**	1.62 (1.16–2.26)	**0.004**
Metabolically healthy with abdominal obesity	412 (37.9)	1.94 (1.58–2.39)	**< 0.001**	1.98 (1.61–2.43)	**< 0.001**	1.89 (1.51–2.36)	**< 0.001**
Metabolically unhealthy with abdominal obesity	306 (28.0)	2.17 (1.71–2.75)	**< 0.001**	2.19 (1.73–2.78)	**< 0.001**	1.74 (1.25–2.42)	**0.001**
Hypertension							
Metabolically healthy without abdominal obesity	563 (28.7)	–	–	–	–	–	–
Metabolically unhealthy without abdominal obesity	299 (48.0)	2.80 (2.15–3.64)	**< 0.001**	2.92 (2.24–3.82)	**< 0.001**	2.49 (1.75–3.52)	**< 0.001**
Metabolically healthy with abdominal obesity	446 (41.0)	2.95 (2.36–3.70)	**< 0.001**	2.97 (2.37–3.73)	**< 0.001**	2.58 (2.02–3.30)	**< 0.001**
Metabolically unhealthy with abdominal obesity	651 (59.7)	5.64 (4.44–7.16)	**< 0.001**	5.67 (4.46–7.20)	**< 0.001**	4.20 (3.02–5.86)	**< 0.001**

WC: waist circumference; OR: odds ratio

Metabolically healthy without abdominal obesity was the reference group

Model 1: adjusted for age and sex;

Model 2: based on model 1 and smoke habits, alcohol consumption, community type, married status and education years

Model 3: based on model 2 and further adjusted for urea, serum uric acid, serum creatinine, fasting plasma glucose, total cholesterol, triglyceride, high-density lipoprotein cholesterol, low-density lipoprotein cholesterol, white blood cell count, red blood cell count, platelet count, hemoglobin A1c, hemoglobin, total protein, albumin, alanine aminotransferase, apolipoprotein A, apolipoprotein B

Significant values was in bold

### Sex-specific association of metabolic obesity phenotype with elevated BP

With regard to metabolic health and abdominal obesity, both men and women with MHAO had higher risks of prehypertension [2.42 (1.52–3.86) in men; 1.76 (1.36–2.29) in women] and hypertension [3.80 (2.38–6.06) in men; 2.22 (1.64–3.00) in women], when compared with those metabolically healthy without abdominal obesity (Table 4). Women with MUGO had nearly five times higher risk of hypertension, compared with those with metabolically healthy without general obesity (Additional file 2: Table S2). Both men and women with MHGO had similar risks of prehypertension and hypertension, compared with those metabolically healthy without general obesity (Additional file 2: Table S2).

**Table 4:** Adjusted odds ratios and 95% confidence intervals of the association of metabolic health and abdominal obesity with prehypertension and hypertension by sex

WC and metabolic status	Men				Women			
	Model 1		Model 2		Model 1		Model 2	
	OR (95% CI)	*P* value	OR (95% CI)	*P* value	OR (95% CI)	*P* value	OR (95% CI)	*P* value
Prehypertension								
Metabolically healthy without abdominal obesity	–	–	–	–	–	–	–	–
Metabolically unhealthy without abdominal obesity	2.05 (1.45–2.89)	**< 0.001**	1.84 (1.18–2.89)	**0.007**	1.22 (0.82–1.82)	0.333	1.36 (0.79–2.36)	0.270
Metabolically healthy with abdominal obesity	2.61 (1.69–4.04)	**< 0.001**	2.42 (1.52–3.86)	**< 0.001**	1.78 (1.39–2.26)	**< 0.001**	1.76 (1.36–2.29)	**< 0.001**
Metabolically unhealthy with abdominal obesity	2.87 (1.89–4.36)	**< 0.001**	2.05 (1.17–3.61)	**0.012**	1.83 (1.35–2.47)	**< 0.001**	1.78 (1.14–2.77)	**0.011**
Hypertension								
Metabolically healthy without abdominal obesity	–	–	–	–	–	–	–	–
Metabolically unhealthy without abdominal obesity	3.39 (2.38–4.84)	**< 0.001**	2.88 (1.82–4.54)	**< 0.001**	2.20 (1.44–3.37)	**< 0.001**	2.39 (1.30–4.37)	**< 0.001**
Metabolically healthy with abdominal obesity	4.16 (2.68–6.45)	**< 0.001**	3.80 (2.38–6.06)	**< 0.001**	2.50 (1.90–3.30)	**< 0.001**	2.22 (1.64-3.00)	**< 0.001**
Metabolically unhealthy with abdominal obesity	5.95 (3.95–8.79)	**< 0.001**	3.83 (2.20–6.66)	**< 0.001**	5.31 (3.91–7.21)	**< 0.001**	4.81 (3.06–7.56)	**< 0.001**

WC: waist circumference; RR: risk ratio

Metabolically healthy without abdominal obesity was the reference group

Model 1: adjusted for age and smoke habits, alcohol consumption, community type, married status and education years

Model 2: based on model 2 and further adjusted for urea, serum uric acid, serum creatinine, fasting plasma glucose, total cholesterol, triglyceride, high-density lipoprotein cholesterol, low-density lipoprotein cholesterol, white blood cell count, red blood cell count, platelet count, hemoglobin A1c, hemoglobin, total protein, albumin, alanine aminotransferase, apolipoprotein A, apolipoprotein B

Significant values was in bold

### Age-specific relationship of metabolic obesity with incident hypertension

We classified subjects into young subjects (aged ≤ 64 years) and elderly subjects (aged ≥ 65 years), as previously reported [24]. As shown in Table 5 and Additional file 3: Table S3, the young subjects with MHO, regardless of general or abdominal obesity, had nearly three times higher risk of incident hypertension after full adjustment than those with MHNO [MHAO: 2.95 (2.29–3.79); MHGO: 3.05 (2.32–4.02)], but this association was not observed in the elderly individuals. Additionally, the ORs of incident hypertension were 5.84 (4.14–8.25) in the young subjects with MUAO, and 5.42 (3.73–7.88) in the young subjects with MUGO, compared with the young subjects with MHAO and MHGO, respectively. The elderly subjects with metabolic unhealthy abdominal obesity had a significantly higher risk of hypertension [OR (95% CI) = 3.02 (1.02–8.95)] (Table 5), but the association was not detected in those with MUGO (Additional file 3: Table S3).

**Table 5:** Adjusted odds ratios and 95% confidence intervals of the association of metabolic health and abdominal obesity with prehypertension and hypertension by different age periods

WC and metabolic status	Yong individuals (18 ≤ age ≤ 64 years old)				Elderly individuals (age ≥ 65 years old)			
	Model 1		Model 2		Model 1		Model 2	
	OR (95% CI)	*P* value	OR (95% CI)	*P* value	OR (95% CI)	*P* value	OR (95% CI)	*P* value
Prehypertension								
Metabolically healthy without abdominal obesity	–	–	–	–	–	–	–	–
Metabolically unhealthy without abdominal obesity	1.74 (1.34–2.27)	**< 0.001**	1.68 (1.19–2.36)	**0.003**	1.61 (0.59–4.39)	0.355	2.04 (0.50–8.40)	0.322
Metabolically healthy with abdominal obesity	2.15 (1.73–2.67)	**< 0.001**	2.05 (1.63–2.59)	**< 0.001**	1.29 (0.63–2.64)	0.493	1.31 (0.56–3.07)	0.539
Metabolically unhealthy with abdominal obesity	2.42 (1.89–3.11)	**< 0.001**	2.01 (1.43–2.83)	**< 0.001**	2.21 (1.02–4.80)	0.045	1.90 (0.52–6.94)	0.330
Hypertension								
Metabolically healthy without abdominal obesity	–	–	–	–	–	–	–	–
Metabolically unhealthy without abdominal obesity	3.09 (2.36–4.06)	**< 0.001**	2.73 (1.91–3.89)	**< 0.001**	4.27(1.72–10.63)	**0.002**	4.71 (1.35–16.48)	**0.015**
Metabolically healthy with abdominal obesity	3.48 (2.76–4.40)	**< 0.001**	2.95(2.29–3.79)	**< 0.001**	1.72 (0.89–3.33)	0.109	1.81 (0.87–3.76)	0.114
Metabolically unhealthy with abdominal obesity	7.44 (5.81–9.54)	**< 0.001**	5.84 (4.14–8.25)	**< 0.001**	4.69(2.25–9.78)	**< 0.001**	3.02 (1.02–8.95)	**0.047**

WC: waist circumference; OR: odds ratio

Metabolically healthy without abdominal obesity was the reference group

Model 1: adjusted for sex and smoke habits, alcohol consumption, community type, married status and education years

Model 2: based on model 2 and further adjusted for urea, serum uric acid, serum creatinine, fasting plasma glucose, total cholesterol, triglyceride, high-density lipoprotein cholesterol, low-density lipoprotein cholesterol, white blood cell count, red blood cell count, platelet count, hemoglobin A1c, hemoglobin, total protein, albumin, alanine aminotransferase, apolipoprotein A, apolipoprotein B

Significant values was in bold

## Discussion

The MHO phenotype has been well established, for its absence of metabolic and cardiovascular complications, but it is debated whether MHO, particularly MHAO, should be intervened to prevent incident hypertension. Based on this nationwide population study, we confirmed the association of MHO phenotype with the risks of prehypertension and hypertension, regardless of general or abdominal obesity.

A recent study has shown that overweight/obesity has a prevalence of 51.2% and hypertension has a prevalence of 27.5% in China, both estimated to reach 70.5% and 35.4% in 2030 [25]. The prevalence of MHO varies, due to the lack of a standardized definition of MHO [26, 27]. The adiposity can be divided into general obesity according to BMI, and abdominal obesity according to WC, and fat deposition in distinct areas employ different mechanisms to induce CVDs [28, 29]. The prevalence rates of MHGO and MHAO were 674 (14.1%) and 1088 (22.8%) in the current study, respectively. Another study based on a rural cohort in central China showed that the prevalence of MHO was 9.3% in people with general obesity and 12.8% in people with abdominal obesity [12]. The individuals in our current study were more representative, because they were enrolled from urban and rural areas from nine provinces across China. Additionally, regardless of general obesity or abdominal obesity, the risk of incident hypertension was four times higher in the subjects with MUO phenotype after full adjustment. This risk is also higher than that in another study based on the 2009–2011 CHNS cohort [30], which may indicate that the risk of hypertension in MUO phenotype increases with age. The ORs of prehypertension in the subjects with MUO phenotype were lower than those in the subjects with MHO phenotype in the current study, possibly because the former cohort are more likely to develop hypertension rather than prehypertension alone.

Our findings showed that the prevalence and risk of hypertension were higher in those with general obesity or abdominal obesity, regardless of metabolic state, compared with those metabolically healthy without obesity. The men with MHAO showed higher ORs of hypertension than women with MHAO in our study. It has been reported that adipose tissue tends to accumulate around the abdomen in men, around the hips and thighs in women [31]. Recent studies have shown that although obesity is a risk factor for adverse events, diminished abdominal fat may reduce obesity-related disorders [32], because less deposition of abdominal fat can increase insulin sensitivity, reduce cardiovascular risks, as well as achieve relatively benign prognosis [33]. Our findings indicated that abdominal adiposity with a healthy metabolic state was associated with a higher adverse cardiometabolic risk in men than in women, which is consistent with that in the previous study [34]. However, this association was contrary when comparing the risks of hypertension between men and the women with MUO, which is consistent with that reported in a 5-year Japanese cohort study [35]. With an unhealthy metabolic state, women have potentially higher contents of brown adipose tissue and pericardial adipose tissue, which is associated with improved cardiometabolic risk [36]. This difference may explain the sex-specific association of hypertension with different metabolic and adiposity states.

The young adults with MHO phenotype aged under 64 years, regardless of general or abdominal adiposity, had significantly higher risks of prehypertension and hypertension than those with MHNO phenotype, but this association did not exist in the elderly with MHNO phenotype. It is well known that aging is another risk factor for CVDs, and the elderly are more likely to have an unhealthy metabolic state. Additionally, the small number of elderly participants with MHO phenotype (MHGO, n = 81; MHAO, n = 158) in this study may bias our finding, which should be verified in a larger-scale elderly cohort study. Our findings indicated that the MHO phenotype, including general and abdominal obesity, was not an absolutely safe condition, and weight loss could reduce the risk of developing elevated BP, which is consistent with other studies [12, 37–39]. Moreover, when comparing the prevalence of prehypertension and hypertension in MUGO and MUAO phenotypes, the subjects with MUO phenotype were more likely to have hypertension rather than prehypertension, suggesting that obesity should be intervened early to prevent subsequent adverse events caused by elevated BP.

The implications of the MHO phenotype are controversial, but it provides a novel concept to focus on the mechanism underlying obesity-related cardiometabolic complications based on lipid accumulation and weight gain without CAs. Compelling evidence has demonstrated the biological mechanisms and phenotypic characteristics of MHO and MUO phenotypes [9, 40, 41]. Abnormal and ectopic fat distribution has been recognized as a prominent determinant of metabolic disorder [42, 43]. Recent studies have reported that ectopic fat distribution and impaired adipose tissue function contribute to insulin resistance, lipotoxicity and inflammatory conditions, all accelerating the development from MHO to MUO [44, 45]. Additionally, distinct signaling molecule signatures associated with MHO, including adiponectin, fibroblast growth Factor 21 and chemerin, have been verified to increased the risk of CVDs [46], indicating that fitness and healthier lifestyle may delay the development of subsequent diseases. The association of hypertension with MHO phenotype may arise from the activation of the renin-angiotensin-aldosterone and sympathetic nervous systems, oxidative stress, and altered cytokines [47, 48]. Metabolomic tools have been used to confirm the role of hepatic and mitochondrial functions in metabolic disturbances, and significant differences in gut microbiota composition have been found between MHO and MUO individuals [49].

## Limitations

This study has several limitations. First, racial homogeneity was a restriction of this study. The CHNS cohort study only represented the Chinese population. Multiple ethnic and large-scale cohort studies are needed to validate our results. The absence of data on fat distribution was another limitation because fat deposition in different parts of the body has a different physiological basis and plays different roles in hypertension. Subjects with hypertension at baseline may exaggerate the predictive value of MUO phenotype for incident hypertension. Additionally, the lack of adjustment for biochemical data at the last visit also challenges the reliability of our findings. Finally, the lack of standardized BP measurements at 1 to 4-week intervals (depending on the BP level) may affect the diagnose of hypertension. Despite these limitations, this study was the first to distinguish abdominal and systemic adiposity combined with metabolically healthy status in the development of prehypertension and hypertension. A further analysis on the effects of sex and age on the relationship between metabolic obesity and elevated BP can make our findings more comprehensive.

## Conclusion

In conclusion, our study added evidence that MHO may be a phenotype of interest in future analysis of hypertension or CVD, which is gleaned from the epidemiologic context. Our findings indicated that the MHO phenotype, regardless of general or abdominal obesity, increased the risks of prehypertension and hypertension, particularly in young adults. Furthermore, metabolically healthy status as a transient state was shown to far increase the risk of hypertension when it changed to a metabolically unhealthy situation. Particular attention should be given to the impact of metabolic state and how it increases the risk of hypertension over time.

## Supplementary Information

**Additional file 1: Table S1.** Comparison of characteristics between target population in 2009 and 2015.

**Additional file 2: Table S2.** Adjusted odds ratios and 95% confidence intervals of the association of metabolic health and general obesity with prehypertension and hypertension by sex.

**Additional file 3: Table S3.** Adjusted odds ratios and 95% confidence intervals of the association of metabolic health and general obesity with prehypertension and hypertension by different age periods.

## Data Availability

All data generated or analysed during this study are included in this published article and its supplementary information files.

## Abbreviations

MHO: Metabolically healthy obesity
MHAO: Metabolically healthy abdominal obesity
MHGO: Metabolically healthy general obesity
CHNS: China Health and Nutrition Survey
CVD: Cardiovascular disease
BMI: Body mass index
CAs: Cardiometabolic abnormalities
WC: Waist circumference
BP: Blood Pressure
SBP: Systolic blood pressure
DBP: Diastolic blood pressure
MAP: Mean arterial pressure
HDL-C: High-density lipoprotein cholesterol
LDL-C: Low-density lipoprotein cholesterol
TC: Total cholesterol
TG: Triglyceride
FPG: Fasting plasma glucose4
ApoA: Apolipoprotein A
ApoB: Apolipoprotein B
NCEP ATP III: National Cholesterol Education Program Adult Treatment Panel III
MUNGO: Metabolically unhealthy non-general obesity
MUGO: Metabolically unhealthy general obesity
MUAO: Metabolically unhealthy abdominal obesity
SD: Standard deviation
OR: Odds ratio
CI: Confidence interval
WBC: White blood cell
RBC: Red blood cell
