# Supplementary material for: Relationship between metabolically healthy obesity and the development of hypertension: a nationwide population-based study
Source: Diabetol Metab Syndr. 2022 Oct 13;14:150. doi: 10.1186/s13098-022-00917-7 (PMC9559015; doi:10.1186/s13098-022-00917-7)
Supplement: Supplementary file 1 — Additional file 1: Table S1. Comparison of characteristics between target population in 2009 and 2015. [file 13098_2022_917_MOESM1_ESM.docx]

| **Table S1.Comparison of characteristics between target population in 2009 and 2015** | | | |
| --- | --- | --- | --- |
| Parameter | Subjects at baseline | Subjects followed up in 2015 | *P* value |
| SBP, mm Hg | 121.33(111.33-136.00) | 129.33(119.33-141.67) | <0.001 |
| DBP, mm Hg | 80.00(72.67-88.00) | 80.67(75.67-89.33) | <0.001 |
| MAP, mm Hg | 94.00(87.33-102.67) | 96.94(90.44-106.00) | <0.001 |
| BMI, kg/m^2^ | 23.29(21.11-25.75) | 23.91(21.71-26.40) | <0.001 |
| WC, cm | 83.00(76.00-90.00) | 85.00(78.00-92.30) | <0.001 |
| Abbreviations: SBP=systolic blood pressure; DBP=diastolic blood pressure; MAP= mean arterial pressure; BMI=body mass index; WC=waist circumference; Continuous variables are shown as mean ± SD if normally distributed or median (quartile 1, quartile 3) if non-normally distributed. The Kruskal-Wallis test was performed. | | | |
