# Supplementary material for: Relationship between metabolically healthy obesity and the development of hypertension: a nationwide population-based study
Source: Diabetol Metab Syndr. 2022 Oct 13;14:150. doi: 10.1186/s13098-022-00917-7 (PMC9559015; doi:10.1186/s13098-022-00917-7)
Supplement: Supplementary file 2 — Additional file 2: Table S2. Adjusted odds ratios and 95% confidence intervals of the association of metabolic health and general obesity with prehypertension and hypertension by sex. [file 13098_2022_917_MOESM2_ESM.docx]

| **Table S2**.Adjusted odds ratios and 95% confidence intervals of the association of metabolic health and general obesity with prehypertension and hypertension by sex | | | | | | | | |
| --- | --- | --- | --- | --- | --- | --- | --- | --- |
| BMI and metabolic status | Men | | | | Women | | | |
|  | Model 1 | | Model 2 | | Model 1 | | Model 2 | |
|  | OR(95%CI) | *P* value | OR(95%CI) | *P* value | OR(95%CI) | *P* value | OR(95%CI) | *P* value |
| Prehypertension |  |  |  |  |  |  |  |  |
| Metabolically healthy without general obesity | — | — | — | — | — | — | — | — |
| Metabolically unhealthy without general obesity | 1.93(1.37-2.72) | **<0.001** | 1.67(1.08-2.59) | **0.022** | 1.30(0.96-1.74) | 0.087 | 1.29(0.87-1.92) | 0.205 |
| Metabolically healthy with general obesity | 1.94(1.28-2.93) | **0.002** | 1.70(1.10-2.63) | **0.018** | 1.84(1.35-2.51) | **<0.001** | 1.72(1.24-2.38) | **0.001** |
| Metabolically unhealthy with general obesity | 2.87(1.88-4.40) | **<0.001** | 1.87(1.05-3.34) | **0.033** | 1.57(1.10-2.24) | **0.013** | 1.42(0.86-2.33) | 0.169 |
| Hypertension |  |  |  |  |  |  |  |  |
| Metabolically healthy without general obesity | — | — | — | — | — | — | — | — |
| Metabolically unhealthy without general obesity | 3.07(2.16-4.37) | **<0.001** | 2.59(1.65-4.08) | **<0.001** | 2.47(1.82-3.37) | **<0.001** | 2.61(1.70-4.00) | **<0.001** |
| Metabolically healthy with general obesity | 3.46(2.29-5.21) | **<0.001** | 3.10(1.99-4.83) | **<0.001** | 3.30(2.38-4.59) | **<0.001** | 3.00(2.10-4.30) | **<0.001** |
| Metabolically unhealthy with general obesity | 6.44(4.25-9.75) | **<0.001** | 4.25(2.39-7.55) | **<0.001** | 5.59(4.00-7.83) | **<0.001** | 5.02(3.08-8.18) | **<0.001** |
| BMI, body mass index; OR, odds ratios.  Metabolically healthy without general obesity was the reference group.  Model 1: adjusted for age and smoke habits, alcohol consumption, community type, married status and education years.  Model 2: based on model 2 and further adjusted for urea, serum uric acid, serum creatinine, fasting plasma glucose, total cholesterol, triglyceride, high-density lipoprotein cholesterol, low-density lipoprotein cholesterol, white blood cell count, red blood cell count, platelet count, hemoglobin A1c, hemoglobin, total protein, albumin, alanine aminotransferase, apolipoprotein A, apolipoprotein B.  Significant values was in bold. | | | | | | | | |
