# Supplementary material for: Relationship between metabolically healthy obesity and the development of hypertension: a nationwide population-based study
Source: Diabetol Metab Syndr. 2022 Oct 13;14:150. doi: 10.1186/s13098-022-00917-7 (PMC9559015; doi:10.1186/s13098-022-00917-7)
Supplement: Supplementary file 3 — Additional file 3: Table S3. Adjusted odds ratios and 95% confidence intervals of the association of metabolic health and general obesity with prehypertension and hypertension by different age periods. [file 13098_2022_917_MOESM3_ESM.docx]

| **Table S3**.Adjusted odds ratios and 95% confidence intervals of the association of metabolic health and general obesity with prehypertension and hypertension by different age periods | | | | | | | | |
| --- | --- | --- | --- | --- | --- | --- | --- | --- |
| BMI and metabolic status | Yong individuals(18≤ age≤64 years old) | | | | Elderly individuals(age≥65 years old) | | | |
|  | Model 1 | | Model 2 | | Model 1 | | Model 2 | |
|  | OR(95%CI) | *P* value | OR(95%CI) | *P* value | OR(95%CI) | *P* value | OR(95%CI) | *P* value |
| Prehypertension |  |  |  |  |  |  |  |  |
| Metabolically healthy without general obesity | — | — | — | — | — | — | — | — |
| Metabolically unhealthy without general obesity | 1.66(1.32-2.09) | **<0.001** | 1.48(1.10-2.00) | **0.010** | 1.81(0.83-3.92) | 0.135 | 1.58(0.59-4.26) | 0.363 |
| Metabolically healthy with general obesity | 1.96(1.52-2.52) | **<0.001** | 1.73(1.32-2.27) | **<0.001** | 1.13(0.46-2.77) | 0.792 | 0.87(0.32-2.38) | 0.785 |
| Metabolically unhealthy with general obesity | 2.25(1.70-2.98) | **<0.001** | 1.68(1.15-2.46) | **0.007** | 1.76(0.73-4.26) | 0.208 | 1.64(0.37-7.24) | 0.516 |
| Hypertension |  |  |  |  |  |  |  |  |
| Metabolically healthy without general obesity | — | — | — | — | — | — | — | — |
| Metabolically unhealthy without general obesity | 3.25(2.57-4.10) | **<0.001** | 2.91(2.13-3.96) | **<0.001** | 3.49(1.69-7.17) | **0.001** | 2.69(1.08-6.75) | **0.035** |
| Metabolically healthy with general obesity | 3.64(2.82-4.70) | **<0.001** | 3.05(2.32-4.02) | **<0.001** | 1.69(0.78-3.69) | 0.187 | 1.67(0.70-4.00) | 0.247 |
| Metabolically unhealthy with general obesity | 7.28(5.58-9.51) | **<0.001** | 5.42(3.73-7.88) | **<0.001** | 4.33(1.96-9.59) | **<0.001** | 3.06(0.92-10.15) | **0.068** |
| BMI, body mass index; OR, odds ratios.  Metabolically healthy without general obesity was the reference group.  Model 1: adjusted for sex and smoke habits, alcohol consumption, community type, married status and education years.  Model 2: based on model 2 and further adjusted for urea, serum uric acid, serum creatinine, fasting plasma glucose, total cholesterol, triglyceride, high-density lipoprotein cholesterol, low-density lipoprotein cholesterol, white blood cell count, red blood cell count, platelet count, hemoglobin A1c, hemoglobin, total protein, albumin, alanine aminotransferase, apolipoprotein A, apolipoprotein B.  Significant values was in bold. | | | | | | | | |
